# Metal‐Organic Frameworks as a Thermal Emitter for High‐Performance Passive Radiative Cooling

**DOI:** 10.1002/smtd.202401141

**Published:** 2024-08-16

**Authors:** Do Van Lam, Dao Thi Dung, Uyen Nhat Trieu Nguyen, Hyun Seok Kang, Byeong‐Soo Bae, Hyeon‐Don Kim, Mikyung Lim, Duckjong Kim, Jae‐Hyun Kim, Seung‐Mo Lee

**Affiliations:** ^1^ National Creative Research Initiative Center for Functionally Antagonistic Nano‐Engineering Department of Mechanical Engineering Korea Advanced Institute of Science and Technology (KAIST) 291 Daehak‐ro, Yuseong‐gu Daejeon 34141 South Korea; ^2^ Korea Institute of Machinery and Materials (KIMM) 156 Gajeongbuk‐ro, Yuseong‐gu Daejeon 34103 South Korea; ^3^ University of Science and Technology (UST) 217 Gajeong‐ro, Yuseong‐gu Daejeon 34113 South Korea; ^4^ Wearable Platform Materials Technology Center (WMC) Department of Materials Science and Engineering Korea Advanced Institute of Science and Technology (KAIST) 291 Daehak‐ro, Yuseong‐gu Daejeon 34141 South Korea; ^5^ School of Mechanical and Aerospace Engineering Gyeongsang National University 501 Jinju‐daero Jinju Gyeongnam 52828 South Korea

**Keywords:** metal‐organic framework, optical property, passive radiative cooling, sub‐ambient cooling, thermal emitter, UiO‐66

## Abstract

Passive radiative cooling represents a transformative approach to achieving sustainable cooling on Earth without relying on energy consumption. In this research, the optical characteristics of five readily accessible metal‐organic frameworks (MOFs): ZIF‐67(Co), MOF‐74(Ni), HKUST‐1(Cu), MOF‐801(Zr), and UiO‐66(Zr) are meticulously explored. The objective is to identify the pivotal factors that influence their ability to facilitate radiative cooling. Through an in‐depth analysis encompassing spectroscopic features, surface texture, and porosity, it is found that the MOFs' cooling efficacy is largely influenced by their optical bandgaps and functional groups, although other factors like chemical composition and structural characteristics remain to be considered. Notably, UiO‐66(Zr) emerged as the standout performer, boasting an impressive solar reflectance of 91% and a mid‐infrared emissivity of 96.8%. Remarkably, a fabric treated with UiO‐66(Zr) achieved a substantial sub‐ambient cooling effect, lowering temperatures by up to 5 °C and delivering a cooling power of 26 W m^−2^ at 300 K. The findings underscore the vast potential of MOFs in offering new opportunities to advance passive radiative cooling technologies, paving the way for their extensive application in this field.

## Graphical abstract

This study explores the optical characteristics of five metal‐organic frameworks (MOFs)—ZIF‐67(Co), MOF‐74(Ni), HKUST‐1(Cu), MOF‐801(Zr), and UiO‐66(Zr)—for radiative cooling. Key factors influencing cooling efficacy are largely optical bandgaps and functional groups. UiO‐66(Zr) excels with 91% solar reflectance and 96.8% mid‐infrared emissivity, achieving sub‐ambient cooling of up to 5 °C and highlighting MOFs' potential in passive cooling technologies.

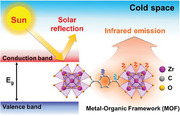

## 1 Introduction

Theoretically, passive daytime radiative cooling (PDRC) can cool a surface for as much as 60 °C below ambient temperature.^[^ ^1^ ^]^ For this reason, PDRC has been considered a sustainable solution to the energy crisis and global warming.^[^ ^2^, ^3^, ^4^ ^]^ Traditional materials, such as TiO_2_,^[^ ^5^ ^]^ SiO_2_,^[^ ^6^, ^7^ ^]^ and Al_2_O_3,_^[^ ^8^ ^]^ have been extensively researched and utilized for PDRC due to their capability to effectively reflect a large portion of sunlight (0.3 ≤ *𝜆* ≤ 2.5 µm) and emit strong thermal radiation in the atmospheric window (AW, 8 ≤ *𝜆* ≤ 13 µm).^[^ ^9^, ^10^ ^]^ Their stability and durability under various environmental conditions have made them popular choices for PDRC applications. To achieve the PDRC effect, the cooler must strongly reflect the solar spectrum to minimize the amount of heat absorbed from the sun.^[^ ^10^, ^11^, ^12^ ^]^ It must also have high thermal emittance in the AW to allow the material to effectively radiate heat in the form of infrared (IR) radiation back into space, bypassing the atmosphere. Consequently, to achieve a PDRC cooler with both high reflectance in the solar spectrum and high emissivity in the AW, ongoing efforts focus on manipulating the electromagnetic properties of the PDRC cooler using approaches such as photonic structures,^[^ ^13^, ^14^, ^15^ ^]^ metamaterials,^[^ ^16^, ^17^, ^18^, ^19^ ^]^ and porous/particle‐embedded polymers.^[^ ^20^, ^21^, ^22^, ^23^, ^24^, ^25^ ^]^ In this context, metal‐organic frameworks (MOFs) have emerged as promising materials for PDRC applications, offering several distinct advantages over traditional materials. First, MOFs provide customizable chemical functionality and tunable porosity,^[^ ^26^ ^]^ enabling precise control over optical and thermal properties. Second, their composition, shape, and size can be tailored through synthetic routes or post‐synthetic modifications,^[^ ^27^, ^28^ ^]^ allowing for properties tailored to specific cooling needs. Third, MOFs demonstrate excellent water adsorption capabilities,^[^ ^29^ ^]^ potentially enhancing cooling through combined radiative and evaporative mechanisms.^[^ ^30^, ^31^ ^]^ Additionally, MOFs are available as micro‐ or nanoparticles,^[^ ^32^ ^]^ making them suitable for use as paint pigments or coating materials on various substrates (Figure S1, Supporting Information). These unique characteristics suggest potential synergies between MOFs and traditional PDRC materials, paving the way for hybrid systems that could significantly improve overall cooling performance. Despite these promising attributes, MOF‐based PDRC applications remain nearly underexplored in the literature.^[^ ^33^, ^34^ ^]^

Here, we have unveiled the extensive and powerful capabilities of MOFs for PDRC applications, marking a significant leap forward in sustainable cooling technologies. We meticulously synthesized and analyzed five prevalent MOFs: ZIF‐67(Co), MOF‐74(Ni), HKUST‐1(Cu), MOF‐801(Zr), and UiO‐66(Zr), focusing on their ability to facilitate efficient radiative cooling. We delved into how the unique spectroscopic features, intricate surface morphology, and precise porous characteristics of these MOFs influence their cooling efficiency when applied as thin films. Our findings revealed a critical insight: the effectiveness of MOFs for radiative cooling is largely influenced by the optical bandgap and functional groups of the MOFs, although other factors like chemical composition and structural characteristics are still non‐negligible. Notably, UiO‐66(Zr) emerged as a standout material, showcasing exceptional solar reflectance of 91% and mid‐infrared emissivity of 96.8%, thus offering superior cooling performance. A fabric coated with UiO‐66(Zr) demonstrated remarkable cooling capabilities, achieving a sub‐ambient temperature reduction of up to 5 °C and a cooling power of 26 W m^−2^ at 300 K.

## 2 Results and Discussion

**Figure 1:**
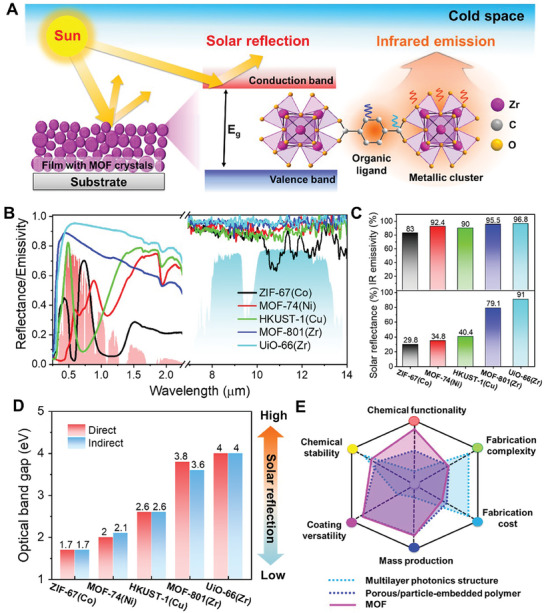
Optical properties of MOFs for radiative cooling. A) Schematic showing the interaction between light and MOF for radiative cooling. B) Profiles showing solar reflectance and IR emissivity of the MOFs in the regions of 0.3–2.5 µm and 7–14 µm, respectively. The solar power density and atmospheric transmittance window are colored red and blue in the background, respectively. C) Averaged IR emissivity (top) and solar reflectance (bottom) of the MOFs, respectively. D) Optical bandgaps of the MOFs determined from Tauc plots. E) Comparison of multilayer photonics structures, porous/particle‐embedded polymers, and MOFs for PDRC applications.

For PDRC, a MOF should be properly chosen to effectively reflect sunlight and radiate heat to the cold space (**Figure** **1**A). Five common MOFs prepared by hydrothermal synthesis were filmed on silicon substrates with a thickness of ≈100 µm to investigate their optical properties. The thickness was purposely chosen for high solar reflectance and mid‐IR emissivity.^[^ ^18^, ^34^ ^]^ The X‐ray diffraction (XRD) patterns of the MOFs were well matched with the corresponding simulated crystal structures of ZIF‐67(Co),^[^ ^35^ ^]^ MOF‐74(Ni),^[^ ^36^ ^]^ HKUST‐1(Cu),^[^ ^37^ ^]^ MOF‐801(Zr),^[^ ^38^ ^]^ and UiO‐66(Zr),^[^ ^39^ ^]^ indicating their successful synthesis (Figure S2, Supporting Information). The high solar reflectance peaks of ZIF‐67(Co) at 0.43 µm, HKUST‐1(Cu) at 0.49 µm, and MOF‐74(Ni) at 0.59 µm accounted for their distinct colors of violet, cyan, and brown, respectively (Figure 1B; Figure S3, Supporting Information). Conversely, MOF‐801(Zr) and UiO‐66(Zr) appeared white due to their high solar reflectance in the whole visible range. High mid‐IR emissivity of all MOFs was consistent with their nature of having abundant IR‐active chemical bonds like C─C, C─O, and metal‐O.^[^ ^4^, ^40^, ^41^ ^]^ The average solar reflectance values of the MOFs varied significantly, ranging from 29.8% to 91% with the sequence ZIF‐67(Co) < MOF‐74(Ni) < HKUST‐1(Cu) < MOF‐801(Zr) < UiO‐66(Zr) (Figure 1C). The optical bandgaps determined from Tauc plots of the five MOFs followed a similar trend to their solar reflectance values (Figure 1D; Figure S4, Supporting Information). Among them, UiO‐66(Zr) showed the highest solar reflectance of 91% and mid‐infrared emissivity of 96.8%. Compared to multilayer optical surfaces^[^ ^8^, ^13^, ^41^ ^]^ and porous/particle‐embedded polymer‐based radiative coolers,^[^ ^4^, ^19^, ^20^ ^]^ utilizing MOFs as PDRC emitters was expected to offer many advantages (Figure 1E). Indeed, MOFs offer superior chemical functionality and excellent chemical stability due to their diverse metal nodes and organic linkers, likely enabling tailored environmental interactions and long‐term performance under a variety of conditions. The versatility of the coatings also allows for easy application to a wide range of substrates and the creation of multifunctional coatings. In addition, MOFs utilize simple and scalable synthesis methods that employ low processing temperatures and inexpensive precursors, potentially offering significant advantages in terms of manufacturing cost and mass production feasibility. Furthermore, MOF synthesis is generally less complex than multilayer photonic structures, potentially resulting in more uniform quality and higher reproducibility. Taken together, these advantages suggest that MOFs have high potential as efficient and cost‐effective materials for radiative cooling applications.

**Figure 2:**
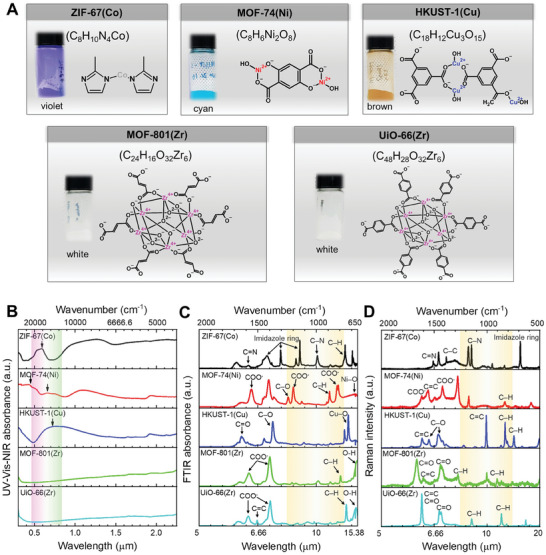
Spectroscopic features of the MOFs. A) Molecular formula and chemical structures of the MOFs. B) UV–vis‐NIR absorbance spectra. The arrows denote visible absorbance peaks of the MOFs. C) ATR‐FTIR absorbance spectra. D) Raman spectra. The yellow column denotes the atmospheric transparent window (8–13 µm).

First of all, spectroscopic characterizations including UV–vis‐NIR diffuse reflectance spectra (UV–vis‐NIR DRS) and attenuated total reflection Fourier transform infrared spectroscopy (ATR‐FTIR) were conducted to evaluate the effects of functional groups (**Figure** **2**A) on the optical properties of the resulting MOFs. The absorption in the NIR spectra is known to arise from the overtone or combination vibrations of H‐containing bonds such as C─H, O─H, N─H, and C═O,^[^ ^42^ ^]^ while the absorption in the UV–vis spectra corresponds to the electronic excitation between the energy levels.^[^ ^43^ ^]^ The presence of visible absorbance peaks in ZIF‐67(Co), MOF‐74(Ni), and HKUST‐1(Cu) (arrows in Figure 2B) accounted for their high solar absorbances. In contrast, the absence of such peaks in MOF‐801(Zr), and UiO‐66(Zr) explained their lower solar absorbances. Furthermore, the absorption peaks in the FTIR spectra are known to correspond to the different vibration modes of functional chemical bonds (Figure 2C).^[^ ^44^ ^]^ Specifically, for ZIF‐67(Co), peaks at 1579, 992, and 756 cm^−1^ are attributed to the vibrations of C═N, C─N, and C─H bonds, respectively; peaks at 1423, 1305, 1143, and 693 cm^−1^ correspond to different vibration modes of the imidazole ring.^[^ ^45^, ^46^ ^]^ For MOF‐74(Ni), peaks at 1553, 1195, and 820 cm^−1^ are due to various vibration modes of COO^−^ bond; peaks at 1241, 888, and 668 cm^−1^ are because of C─O, C─H, and Ni─O bonds, respectively.^[^ ^47^, ^48^ ^]^ For HKUST‐1(Cu), peaks at 1635, 1373, and 729 cm^−1^ indicate the vibrations of C═O, C─O, and Cu─O bonds, respectively.^[^ ^49^, ^50^ ^]^ For MOF‐801(Zr), peaks at 1575 and 1400 cm^−1^ represent the vibration modes of COO^−^ bond; peaks at 795 and 650 cm^−1^ are ascribed to the vibrations of C─H and O─H bonds.^[^ ^51^ ^]^ For UiO‐66(Zr), peaks at 1580 and 1394 cm^−1^ indicate the vibration modes of COO^−^ bond; peaks at 1505, 749, and 662 are originated from C═C, C─H, and O─H bonds, respectively.^[^ ^52^, ^53^ ^]^ Because MOF‐801(Zr) and UiO‐66(Zr) exhibited high emissivity in the wavelength region of 8–13 µm (Figure 1C), we expected to observe a strong IR absorption of those MOFs. However, as compared to other MOFs, the absorption was observed to be less significant, against our expectations. To further investigate, we conducted Raman spectroscopy analysis on these MOFs (Figure 2D), particularly focusing on UiO‐66(Zr). Interestingly, while the FTIR spectra showed less pronounced absorption in the 8–13 µm range, the Raman spectra revealed two notable bending vibrations of the aromatic C─H band within this window.^[^ ^54^ ^]^ FTIR spectroscopy is particularly sensitive to vibrations of polar bonds that involve a change in dipole moment (e.g., asymmetric stretching and bending vibrations of polar C═O, C─O, and O─H bonds), while Raman spectroscopy is more sensitive at detecting vibrations that alter polarizability of the molecule (e.g., symmetric stretching vibrations of non‐polar C═C and C─C bonds and bending vibrations of aromatic C─H bonds).^[^ ^55^ ^]^ Therefore, it was thought that the presence of these bending vibrations of the aromatic C─H bonds, detected in Raman spectroscopy but not in FTIR, is one of the main factors leading to the high emissivity of UiO‐66(Zr) in the 8–13 µm range.

**Figure 3:**
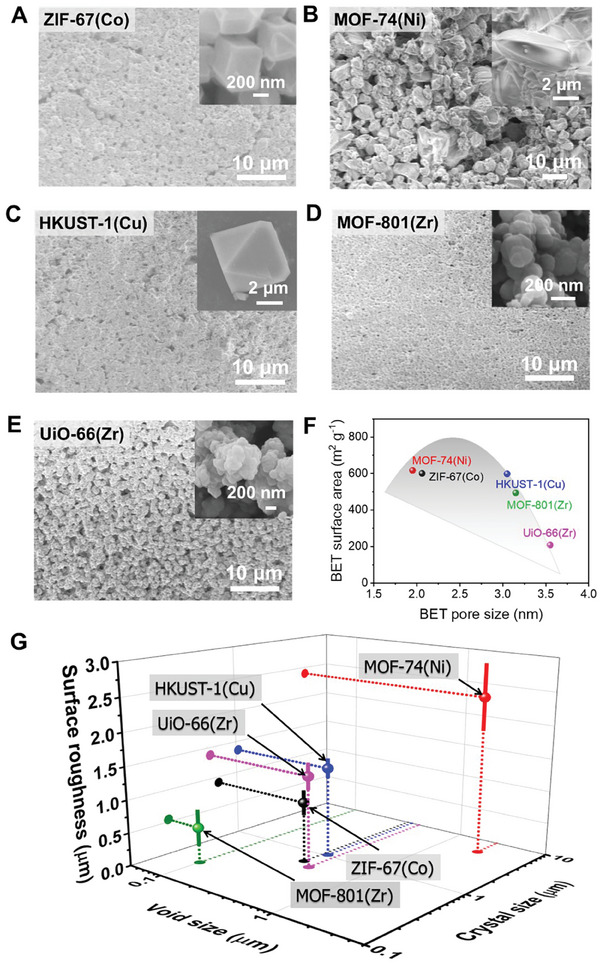
Surface morphology and porous characteristics of the MOFs. A–E) SEM images showing the surface morphology of the MOF films. The inset is the representative crystal of each MOF. F) Plot showing the relationship between BET pore size and BET surface area of the MOFs. G) Plot showing the relationship between void size, crystal size, and surface roughness of the MOF films.

Particle size, pore size, and porosity are known to be also essential factors in determining the optical properties of a PDRC emitter.^[^ ^20^, ^21^, ^56^ ^]^ When the dimension of the pores matches the wavelength of the incident light, multiple scattering at the air/pore interface enhances the light reflectance significantly.^[^ ^10^ ^]^ Pores with diameters ranging from tens, hundreds, to thousands of nanometers exhibit high efficiency in scattering UV, visible, and IR radiation, respectively.^[^ ^56^ ^]^ Particle size also plays an equally important role as the pore size in particle‐embedded coolers. In the case of porosity, it has an inverse correlation with IR emissivity. Namely, high porosity generally leads to a sharp decrease in IR emissivity.^[^ ^56^ ^]^ For these reasons, we examined the porosity and surface morphology of the MOFs. Morphology analysis using scanning electron microscopy (SEM) showed that each MOF crystal has unique particle shapes (**Figure** **3**A–E). The porosity of each MOF was examined by Brunauer–Emmett–Teller (BET) measurements. Specific surface areas (SSAs) of the MOFs were found to range from 200 to 620 m^2^ g^−1^ (Figure S5, Supporting Information). The estimated average pore sizes were 2–3.5 nm, which was two order‐of‐magnitude shorter than the sunlight wavelengths. The SSA and pore size of MOFs appeared to be in inverse relation (Figure 3F). However, it seemed that there is no direct correlation between the SSA and pore size of the MOFs and their solar reflectance and IR emissivity (Figure 1B,C). The carefully measured sizes of each MOF crystal and void were in the range of hundreds of nanometers to several micrometers (Figure S6, Supporting Information). Although ZIF‐67(Co) and UiO‐66(Zr) exhibited similar crystal and void sizes (ZIF‐67(Co): 0.66 ± 0.18 µm and 0.33 ± 0.13 µm; UiO‐(66(Zr): 0.56 ± 0.12 µm and 0.42 ± 0.14 µm), ZIF‐67(Co) shows relatively lower solar reflectance as well as IR emissivity as compared to UiO‐66(Zr). Finite‐difference time‐domain (FDTD) simulations suggested that differences in the particle and void sizes could impact the optical properties of UiO‐66(Zr) (Figure S7, Supporting Information). However, our experimental results indicated that the sizes of crystals and voids are less decisive in determining the solar reflectance and IR emissivity of the five MOFs.

Because the data so far explored did not show clear evidence that solar reflectance and IR emissivity of MOF film is dependent on SSA, pore size, and size of void/crystal for the five MOFs, we considered other factors, i.e., surface roughness (Figure S8, Supporting Information). The relation between surface roughness and reflectance has long been studied and formulated using the concept of total integrated scatter (TIS) as follows:^[^ ^57^, ^58^, ^59^ ^]^

(1) $$TIS\;=\frac{diffuse\;reflectance}{total\;reflectance}\;=\frac{R_{d}}{R_{t}}\;=\;1-\exp\left[-{\left(4\pi cos\theta_{i}\frac{\sigma }{\lambda }\right)}^{2}\right]$$

(2) $$R_{d}=R_{t}\;\left[1-\exp\left[-{\left(4\pi cos\theta_{i}\frac{\sigma }{\lambda }\right)}^{2}\right]\right]$$

where

(3) $$\sigma \;=\sqrt{\frac{1}{L}\int_{0}^{L}y^{2}\left(x\right)dx}\;$$

In these equations, *y*(*x*),  λ, σ, *L*, and θ_*i*_ are the surface profile, the wavelength of light, the root mean square (rms) surface roughness, the assessment length, and the angle of incidence on the surface, respectively. Equation (2) tells that i) the diffuse reflectance is proportional to the surface roughness, ii) light with a shorter wavelength reflects more than light with a longer one, and iii) more light reflects at normal incidence than grazing incidence. It is also known that surface roughness can affect the thermal emissivity of a material, although the relationship between them is not strictly proportional in a linear sense.^[^ ^60^ ^]^ The emissivity was reported to be also dictated by internal reflections within the slope of the valleys that are created by the surface features.^[^ ^61^ ^]^ Wen and Mudawar proposed three emissivity regimes based on the characteristic ratio of σ/λ: i) optically smooth surface (σ/λ ≪ 1); ii) intermediate region (0.2 < σ/λ < 1); iii) geometric region (σ/λ > 1).^[^ ^62^ ^]^ They mentioned that surface morphology (the slope of the peaks and valleys of the surface) can play a key role in the emissivity trend when σ/λ > 1. Taking into account this fact and a previous study,^[^ ^63^ ^]^ we initially expected that UiO‐66(Zr) has a higher value of σ/λ than that of ZIF‐67(Co) in the IR region, thereby exhibiting higher IR emissivity. However, MOFs with even higher roughness (e.g., MOF‐74(Ni) (Figure 3G) showed varied solar reflectance and IR emissivity, indicating complexity in how surface texture impacts optical behavior.

As a next analysis, we revisited the colors of MOFs themselves being displayed under visible light and their optical bandgaps (E_g_), because there is a clear correlation between the E_g_ and its visible color.^[^ ^64^ ^]^ The bandgap represents the minimum energy of light that the material can absorb, which corresponds to a specific wavelength of light. When a material absorbs light at this wavelength, it becomes invisible, and the light reflected or transmitted at other wavelengths determines the color of the material. For MOFs, it is known that the size of the bandgap determines the range of light they can absorb, which in turn affects the color they display.^[^ ^65^, ^66^ ^]^ So, we hypothesized that a MOF with a large bandgap might absorb higher energy light (such as UV) and reflect visible light more, appearing transparent or white. Conversely, we guessed that a MOF with a narrow bandgap might absorb some of the visible light, displaying the complementary color of the absorbed wavelengths. We figured out that white‐colored MOFs (UiO‐66(Zr) and MOF‐801(Zr)) have relatively wider E_g_ values than the other non‐white‐colored MOFs (HKUST‐(Cu), MOF‐74(Ni), and ZIF‐67(Co), i.e., direct E_g_, _UiO‐66(Zr)_ = 4.0 eV > E_g_, _MOF‐801(Zr)_ = 3.8 eV > E_g_, _HKUST‐1(Cu)_ = 2.6 eV > E_g_, _MOF‐74(Ni)_ = 2.0 eV > E_g, ZIF‐67(Zn)_ = 1.7 eV (Figure 1D).

From the data so far obtained (**Table** **1**), we were able to conclude that the optical bandgap value of MOF is likely one of the pivotal factors in determining the PDRC performance of the MOF. Indeed, MOFs with wide E_g_ (such as MOF‐801(Zr) and UiO‐66(Zr)) generally exhibited superior solar reflectance and IR emissivity. The C–H vibrations also seemed to partly enhance the IR emissivity of the MOFs. This suggested that the ability to scatter a broad spectrum of light and effectively emit IR radiation is closely tied to the E_g_ of MOF and vibrations of functional groups. The relationship between BET surface area, pore size, particle size, void size, surface roughness, and optical properties of MOFs exhibited a less straightforward trend, pointing to a complex interplay of factors. These findings underscored the complexity of the relationship between MOFs' structural characteristics and their optical properties. The bandgap and functional groups were observed to correlate with both solar reflectance and IR emissivity. However, a comprehensive understanding of each factor's impact, particularly the less clear role of particle size, void size, and surface roughness, remains to be evaluated further.

**Table 1:** Overview of MOF optical and physical characteristics.

Feature/MOFs	ZIF‐67 (Co)	MOF‐74 (Ni)	HKUST‐ 1 (Cu)	MOF‐801 (Zr)	UiO‐66 (Zr)
Visible color	violet	brown	cyan	white	white
Optical band gap (eV) from Tauc direct bandgap	1.7	2.0	2.6	3.8	4.0
Optical bandgap (eV) from Tauc indirect bandgap	1.7	2.1	2.6	3.6	4.0
Solar reflectance (%) in wavelength 0.3–2.5 µm	29.8	34.8	40.4	79.1	91
IR emissivity (%) in wavelength 8–13 µm	83	92.4	90	95.5	96.8
BET surface area (m^2^ g^−1^)	601	617	598	494	208
BET pore size (nm)	2.1	1.9	3.1	3.2	3.6
MOF particle size (µm)	0.66	5.58	1.05	0.12	0.56
Void size between MOF particles (µm)	0.33	2.03	0.36	0.1	0.42
Surface roughness (µm)	0.91	2.40	1.36	0.54	1.38

**Figure 4:**
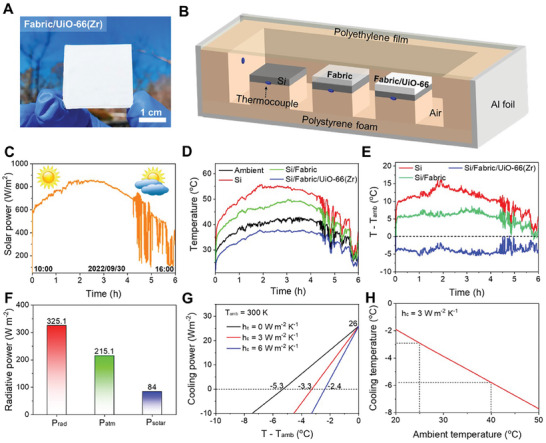
Radiative cooling performances of the UiO‐66(Zr). A) A digital image showing a UiO‐66(Zr)‐coated polyester fabric for radiative cooling. B) Schematic illustration of the thermal box setup for radiative cooling measurement. C–E) Solar power profile, temperature profiles, and temperature difference (T – T_amb_) profiles recorded in the daytime from 10:00 to 16:00 on 30 Sep 2022, respectively. F) Radiative powers of the radiative cooler. G) The net cooling power of the sample. H) Cooling temperature as a function of ambient temperature.

To demonstrate a practical application of the MOFs for PDRC, UiO‐66(Zr) was selected to conduct the outdoor radiative cooling experiments. UiO‐66(Zr) was coated onto a polyester fabric by screen printing (i.e., fabric/UiO‐66(Zr)) (**Figure** **4**A). Morphological changes on the fabric before and after the UiO‐66(Zr) coating were shown in Figure S9 (Supporting Information). It was found that the radiative cooler with a thickness of 640 µm or higher can ensure low solar transmittance of less than 1% and eliminates the need for metal reflectors used in photonic structures or thin, porous polymers (Figure S10, Supporting Information).^[^ ^8^, ^14^, ^67^ ^]^ The optical solar absorptivity and the IR emissivity of the emitter were displayed in Figure S11 (Supporting Information). The prepared fabric/UiO‐66(Zr) cooler was loaded inside a thermal box made of thermally insulative polystyrene foam covered with a reflective Al foil and a transparent low‐density polyethylene window (Figure 4B). The thermal box was placed onto the top of a one‐meter‐high utility cart to avoid heat conduction from the ground (Figure S12, Supporting Information). This setup was specifically designed to minimize external factors such as wind fluctuations and humidity variations which can significantly impact cooling performance measurements. The closed system allowed us to maintain more consistent and controlled conditions which are necessary for assessing the intrinsic cooling capabilities of our MOF samples more objectively.

The experiment was conducted during daylight hours on an autumn day in Daejeon, South Korea. The air humidity during the test ranged from 35–40%. Si wafer and bare fabric were used as references for comparison. The weather was clear from 10:00 to 14:30 and sometimes cloudy after that (Figure 4C). From 11:30 to 13:30, the solar power was over 800 W m^−2^ and reached the highest of 850 W m^−2^ at noon. Because of the top‐covered low‐density polyethylene film, the greenhouse effect caused the temperature inside the thermal box to be hot (Figure 4D), likely due to the nonideal transmittance of the film.^[^ ^8^ ^]^ During the clear‐sky hours, the fabric/UiO‐66(Zr) maintained a steady‐state cooling temperature of 4–5 °C below the ambient air, while the Si wafer and the bare fabric maintained 12–15 °C and 6–7 °C above the ambient air, respectively (Figure 4E). During the cloudy condition, a sub‐ambient cooling temperature of ≈2 °C was still observed, likely due to the reduced transparency of the atmospheric transparent window.^[^ ^10^ ^]^ The cooling capability was highly comparable to other reported PDRC emitters in the literature (Table S1, Supporting Information). Additionally, the solar reflectance of the fabric/UiO‐66(Zr) cooler was observed to be nearly unchanged even after over a week of exposure to natural light (Figure S13, Supporting Information). The adhesion between UiO‐66(Zr) and substrate was found to be rather stable although it largely depended on the binder (Figure S14, Supporting Information). We also checked the thermal conductivity of UiO‐66(Zr) because materials with lower thermal conductivity can maintain a temperature difference with their surroundings more effectively. The experimentally measured thermal conductivity of UiO‐66(Zr) was reported to be 0.11 W m^−1^ K^−1^.^[^ ^68^ ^]^ It was lower than the other MOFs like HKUST‐1(Cu)^[^ ^69^ ^]^ with 0.69 W m^−1^ K^−1^ and MOF‐74(Zn)^[^ ^70^ ^]^ with 0.44 W m^−1^ K^−1^. The low conductivity of UiO‐66(Zr) was likely one of the factors contributing to its high cooling capability.

To analyze the theoretical cooling power of the UiO‐66(Zr)‐based emitter, an energy balance model was employed.^[^ ^8^, ^13^ ^]^ When a surface with temperature T is exposed to an environment with an ambient temperature T_amb_, the theoretical net cooling power (P_net_) is given by:

(4) $$P_{net}\;\left(T\right)=P_{rad}\;\left(T\right)-\;P_{atm}\left(T_{amb}\right)-\;P_{solar}-P_{non-rad}\left(T,T_{amb}\right)$$

where *P_rad_* (*T*), *P_atm_* (*T_amb_*), *P_solar_*, and  *P*_*non* − *rad*_(*T*, *T_amb_*) are the power radiated by the cooler surface, the absorbed atmospheric radiation power, the absorbed solar radiation power, and the non‐radiative heat loss contributed by natural convection and conduction, respectively. For our study, we assumed a model where the radiative heat transfer only occurs in the solar spectrum (0.3 –2.5 µm) and part of the mid‐IR wavelengths (4–20 µm). A standard mid‐latitude winter was used to model the atmospheric transmittance using MODTRAN web app. Based on this model, on a clear‐sky day at T_amb_ = 27 °C (300 K), P_rad_, P_atm_, and P_solar_ were calculated to be 325.1, 215.1, and 84 W m^−2^, respectively (Figure 4F). Therefore, the P_net_ was 26 W m^−2^ with a maximum temperature drop of 5.3 °C (Figure 4G). Notably, UiO‐66(Zr) was the only MOF among the MOFs that demonstrates a positive theoretical cooling power. The negative values observed for the other MOFs suggested that these materials would absorb more energy than they emit, resulting in net heating rather than cooling (Figure S15, Supporting Information). When the natural‐convection heat transfer coefficient *h_c_* of 3 W m^−2^ K^−1^ was used for the P_non‐rad_, a cooling temperature of 3.3 °C below the ambient was able to be achieved. With increasing the ambient temperature to 40 °C, the temperature drop increased to ≈5.6 °C (Figure 4H), which was consistent with the experimental cooling temperature.

## 3 Conclusion

In summary, we demonstrated that MOFs can be widely used for PRDC applications. Through the comparative study, we found that the MOFs' cooling efficacy is largely influenced by their optical bandgaps and functional groups, although other factors like chemical composition, and structural characteristics remain to be considered. However, achieving a nuanced understanding of each factor's contribution to these properties required more in‐depth research and analysis. Among the synthesized MOFs, UiO‐66(Zr) films exhibited high solar reflectance of 91% and mid‐IR emissivity of 96.8%. In addition, the UiO‐66(Zr)‐based fabric showed a high sub‐ambient cooling temperature of up to 5 °C and a cooling power of 26 W m^−2^ at 300 K. Because thousands of MOFs remain unexplored, and new MOFs are continuously displaying in the literature, it is strongly believed that MOFs outperforming the current mainstream materials for radiative cooling applications will soon appear in the market.

## Conflict of Interest

The authors declare no conflict of interest.

## Supporting information

Supporting Information

## Data Availability

The data that support the findings of this study are available in the supplementary material of this article.
