# Supplementary material for: Metal‐Organic Frameworks as a Thermal Emitter for High‐Performance Passive Radiative Cooling
Source: Small Methods. 2024 Aug 16;9(3):2401141. doi: 10.1002/smtd.202401141 (PMC11926517; doi:10.1002/smtd.202401141)
Supplement: Supplementary file 1 — Supporting Information [file SMTD-9-2401141-s001.pdf]

small
methods

Supporting Information

for *Small Methods*, DOI 10.1002/smtd.202401141

Metal-Organic Frameworks as a Thermal Emitter for High-Performance Passive Radiative Cooling

*Do Van Lam, Dao Thi Dung, Uyen Nhat Trieu Nguyen, Hyun Seok Kang, Byeong-Soo Bae, Hyeon-Don Kim, Mikyung Lim, Duckjong Kim, Jae-Hyun Kim and Seung-Mo Lee**

Supporting information for

Metal-Organic Frameworks as a Thermal Emitter for High-Performance Passive Radiative Cooling

Do Van Lam,[a] Dao Thi Dung,[b,c] Uyen Nhat Trieu Nguyen,[b,c] Hyun Seok Kang,[d] Byeong-Soo Bae,[d] Hyeon-Don Kim,[b] Mikyung Lim,[b] Duckjong Kim,[e] Jae-Hyun Kim,[b,c] Seung-Mo Lee[b,c,*]

[a] National Creative Research Initiative Center for Functionally Antagonistic Nano-Engineering, Department of Mechanical Engineering, Korea Advanced Institute of Science and Technology (KAIST), 291 Daehak-ro, Yuseong-gu, Daejeon, 34141, South Korea

[b] Korea Institute of Machinery and Materials (KIMM), 156 Gajeongbuk-ro, Yuseong-gu, Daejeon 34103, South Korea.

[c] University of Science and Technology (UST), 217 Gajeong-ro, Yuseong-gu, Daejeon 34113, South Korea.

[d] Wearable Platform Materials Technology Center (WMC), Department of Materials Science and Engineering, Korea Advanced Institute of Science and Technology (KAIST), 291 Daehak-ro, Yuseong-gu, Daejeon, 34141, South Korea

[e] Department of Mechanical Engineering, Gyeongsang National University, 171 Jang-dong, Yousung-gu, Jinju 52828, South Korea

* Correspondence to: S.M.L. (sm.lee@kimm.re.kr)

Experimental section

Synthesis of MOFs

To investigate the radiative properties, five commonly used MOFs, including ZIF-67(Co), HKUST-1(Cu), MOF-74(Ni), MOF-801(Zr), and UiO-66(Zr), were synthesized by the hydrothermal process as follows: (See Table S2 for detailed synthetic conditions)

(1) ZIF-67(Co): $Co(NO_3)_2 \cdot 6H_2O$ (3.321 g, 13.3 mmol) and 2-methyl imidazole ($C_4H_6N_2$; 3.284 g, 40 mmol) were dissolved in 100 mL ethanol (EtOH, C_2H_5OH) by sonication for 30 min. The solution was transferred into a Teflon-lined autoclave and then heated in an electric oven for 24 h at 120 °C. After cooling to room temperature, violet crystals were collected by centrifugation, washed with methanol, and then dried naturally in the air overnight.

(2) MOF-74(Ni): A solution of $Ni(NO_3)_2 \cdot 6H_2O$ (0.600 g, 2.063 mmol) and 2,5-dihydroxyterephthalic acid (H_4DOBDC, $C_6H_8O_6$; 0.118 g, 0.590 mmol) dissolved in 2 mL N,N-diethyl formamide (DEF, $HCON(C_2H_5)_2$) and another solution of melamine ($C_3N_3(NH_2)_3$; 0.100 g, 0.793 mmol) dissolved in 10 mL ethylene glycol (EG, $(CH_2OH)_2$) was prepared by sonication for 1 h. The two solutions were mixed rigorously and then transferred into a Teflon-lined autoclave. The MOF synthesis was conducted for 24 h at 130 °C. After cooling to room temperature, brown crystals were collected by filtration, washed with methanol, and then dried naturally in the air overnight.

(3) HKUST-1(Cu): A solution of $Cu(NO_3)_2 \cdot 3H_2O$ (2.585 g, 10.7 mmol) dissolved in 30 mL H_2O and another solution of trimesic acid (H_3BTC, $C_9H_6O_6$; 0.672 mg, 3.2 mmol) dissolved in 30 mL ethanol was prepared by sonication for 30 min, respectively. The two solutions were slowly mixed and thoroughly stirred until becoming turbid. 2 mL dimethylformamide (DMF, C_3H_7NO) as the crystallizing solvent was then added to the solution and continuously stirred for 5 min. The prepared solution was transferred into a Teflon-lined autoclave and heated in the electric oven for 20 h at 80 °C. After cooling

to room temperature, cyan crystals were collected by filtration, washed with water and ethanol, and then dried naturally in the air overnight.

(4) MOF-801(Zr): $ZrCl_4$ (0.120 g, 0.516 mmol), fumaric acid ($C_2H_2(COOH)_2$; 0.180 g, 1.552 mmol), and benzoic acid (C_6H_5COOH; 3.503 g, 28.69 mmol) were dissolved in 20 mL DMF by sonication for 1 h. The homogenous solution was transferred to a Teflon-lined autoclave, and the MOF synthesis was conducted for 24 h at 120 °C in an electric oven. After cooling to temperature, white crystals were collected by centrifugation at 7500 rpm for 5 min 3 times in DMF and dried naturally in the air overnight.

(5) UiO-66(Zr): $ZrCl_4$ (0.080 g, 0.37 mmol) was dissolved in a 10 mL DEF solution, and terephthalic acid (H_2BDC, $C_6H_4(COOH)_2$; 0.050 g, 0.3 mmol) was dissolved in another 10 ml DEF solution by sonication for 1 h. The two solutions were mixed and stirred for 10 min. 2 mL formic acid (HCOOH) was added to the solution to modulate the crystal formation and growth. The resulting solution was sealed in a Teflon-lined autoclave, and the MOF synthesis was carried out for 48 h at 135 °C in an electric oven. White crystals were collected by centrifugation at 7500 rpm for 15 min 3 times in methanol and dried naturally in the air overnight.

Optical characterizations

MOF films were prepared by grinding 100 mg MOF and 10 mg polyvinylidene fluoride (PVDF) in a mortar with a proper amount of N-methyl-2-pyrrolidone (NMP) solvent for 10 min to form a homogeneous slurry and followed by casting the slurry onto a 2 x 2-cm^2 Si wafer. The films were then annealed at 70 °C overnight before optical characterizations. The spectral reflectance $\rho(\lambda)$ and transmittance $\tau(\lambda)$ in the solar spectrum range (0.3 – 2.5 μm) were measured using a UV-VIS-NIR spectrophotometer (UV-3600 plus, Shimadzu) equipped with an integrating sphere. The spectral emissivity in the mid-IR range (2.5 – 14 μm) was measured using an FTIR spectroscopy (FTLA 2000 series, ABB) equipped with an integrating sphere. UV-VIS-NIR DRS (V-770, JASCO International Co., Ltd.) and ATR-FTIR (Nicolet 6700, Thermo Fisher Scientific) were used to characterize the solar and thermal absorbance spectra

of the MOFs and their precursors, respectively. The average solar reflectance (R_{solar}) and IR emissivity (ε_{IR}) were calculated using the following formula:[S1]

$$R_{solar} = \int_{0.3}^{2.5} I_{AM1.5}(\lambda)\rho(\lambda)d\lambda \frac{1}{\int_{0.3}^{2.5} I_{AM1.5}(\lambda)d\lambda}$$

and

$$\varepsilon_{IR} = \int_{8}^{13} I_{BB}(T,\lambda)\varepsilon(T,\lambda)d\lambda \frac{1}{\int_{8}^{13} I_{BB}(T,\lambda)d\lambda}$$

where $I_{AM1.5}(\lambda)$ and $I_{BB}(T,\lambda)$ are the solar radiation with AM1.5 filter and the blackbody radiation at temperature T, respectively. For diffuse surfaces, at thermal equilibrium, the spectral emissivity ε(λ) is equal to the absorptivity α(λ) according to Kirchhoff's law:

$$\varepsilon(\lambda) = 1 - \rho(\lambda) - \tau(\lambda).$$

Fabrication of UiO-66(Zr)-painted fabric

A 3 x 3 cm^2 piece of polyester fabric was used as a substrate for painting UiO-66(Zr). Initially, 200 mg UiO-66(Zr) and 30 mg PVDF were ground in a mortar with a proper amount of NMP solvent for 10 min to form a homogeneous slurry. The slurry was then painted onto the fabric by screen-printing. The fabric/UiO-66(Zr) was obtained by heating the sample on a hotplate at 70 °C overnight. The fabric/UiO-66(Zr) emitter has a thickness of ~220 µm. A multilayer of fabric/UiO-66(Zr) structure was used to prepare a thicker emitter.

Materials characterization

Morphology and elemental composition were characterized using SEM (JSM-700F, JEOL) equipped with EDX (AZtec X-Max, Oxford instruments). The phase structure and functional chemical bonds were examined by XRD (Cu Kα, Empyrean, Panalytical), Raman microscopy (λ = 514 nm, inVia, Renishaw), and FTIR instrument (Thermal Scientific) equipped with an attenuated total reflection (ATR) accessory. The particle size and micropore size of each MOF were roughly calculated from SEM images using Gatan Microscopy Suite software. The surface roughness of the MOF films was characterized by confocal microscopy.

Outdoor passive radiative cooling measurements

A system for outdoor radiative cooling experiments was built with a thermal box, a solar power meter, a temperature sensor, and a laptop. The thermal box consisted of a thermally insulative polystyrene foam covered with a reflective Al foil. A transparent low-density polyethylene film was used to seal the thermal box and served as a windshield. The thermal box was placed onto the top of a 1 m height utility cart to avoid heat conduction from the ground. The MOF-based fabric was placed onto a 2 x 2-cm^2 Si wafer mounted with a K-type temperature sensor on the back side to detect the real-time temperature of the sample. The temperature was recorded by a laptop-connected data-logging thermometer with an uncertainty of 0.1 °C (Center309, CENTER). An ambient thermocouple was placed drop-down at the edge side of the apparatus to minimize the direct solar heating and to detect real-time in-apparatus temperature. The solar irradiation was recorded using a data-logging solar power meter with an accuracy of 5% (TES132, TES Electrical Electronic Corp.) As a control, the polyester fabric and Si wafer were also mounted in the same apparatus to compare the cooling performances. The experiment was conducted on a flat surface at the Korea Institute of Machinery & Materials, Daejeon, South Korea, in September 2022.

FDTD simulations for UiO-66(Zr)

For FDTD simulation, the refractive index (*n*) and extinction coefficient (κ) of UiO-66(Zr) were assumed to be ~1.2 and 0, based on the previous study[S2] and the low solar absorption of the MOF. The MOF particles with an average diameter of ~0.56 μm were assumed to have spherical shapes. The porosity (ϕ) of the MOF film was calculated based on its density as below:

$$\rho_{film} = m_{film}/V_{film}$$

$$\phi = 1 - \rho_{film}/\rho_{theory}$$

where m_{film}, V_{film}, and ρ_{film} are the actual mass, volume, and density of the UiO-66(Zr) film, respectively, while ρ_{theory} = 1.237 g cm^{-3} is the theoretical density of the UiO-66(Zr) based on

the literature.[S3] According to our measurement, ρ_{film} = 0.743 g cm^{-3}, so the porosity value of the UiO-66(Zr) film was ~40%. The influence of the MOF particle and void sizes on the scattering properties of the UiO-66(Zr) film was investigated using Lumerical FDTD Solutions software.[S4,5] A simple two-dimensional model was used to simplify the analysis and to reduce the simulation time. A perfect matching layer absorption boundary conditions and a full field scattering field source were used.[S4] The scattering efficiencies of the particles were simulated in the solar wavelength range of 0.3 – 2.5 μm.

Supporting figures

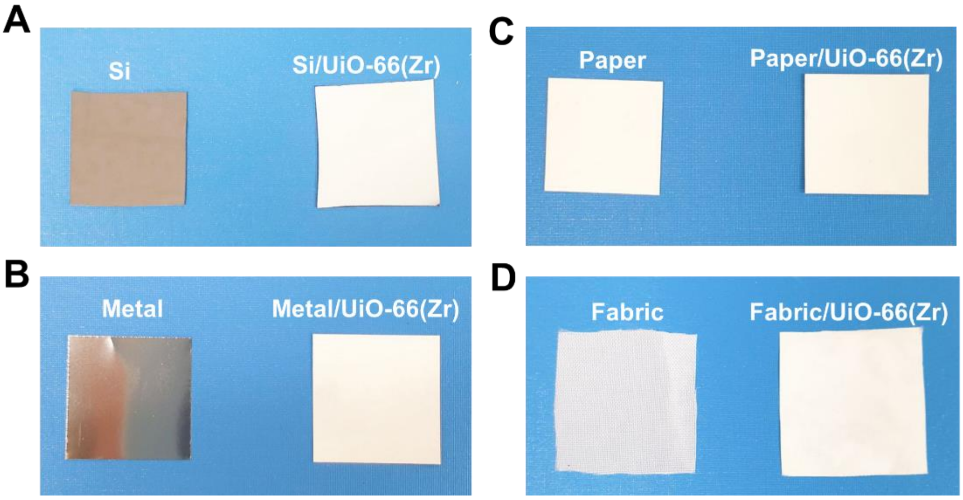

Figure S1. Coating versatility of UiO-66(Zr) onto various substrates: (**A**) silicon, (**B**) metal, (**C**) paper, and (**D**) fabric. The coating process involved preparing a homogeneous slurry of UiO-66(Zr) and applying it to the substrates via a screen-printing method.

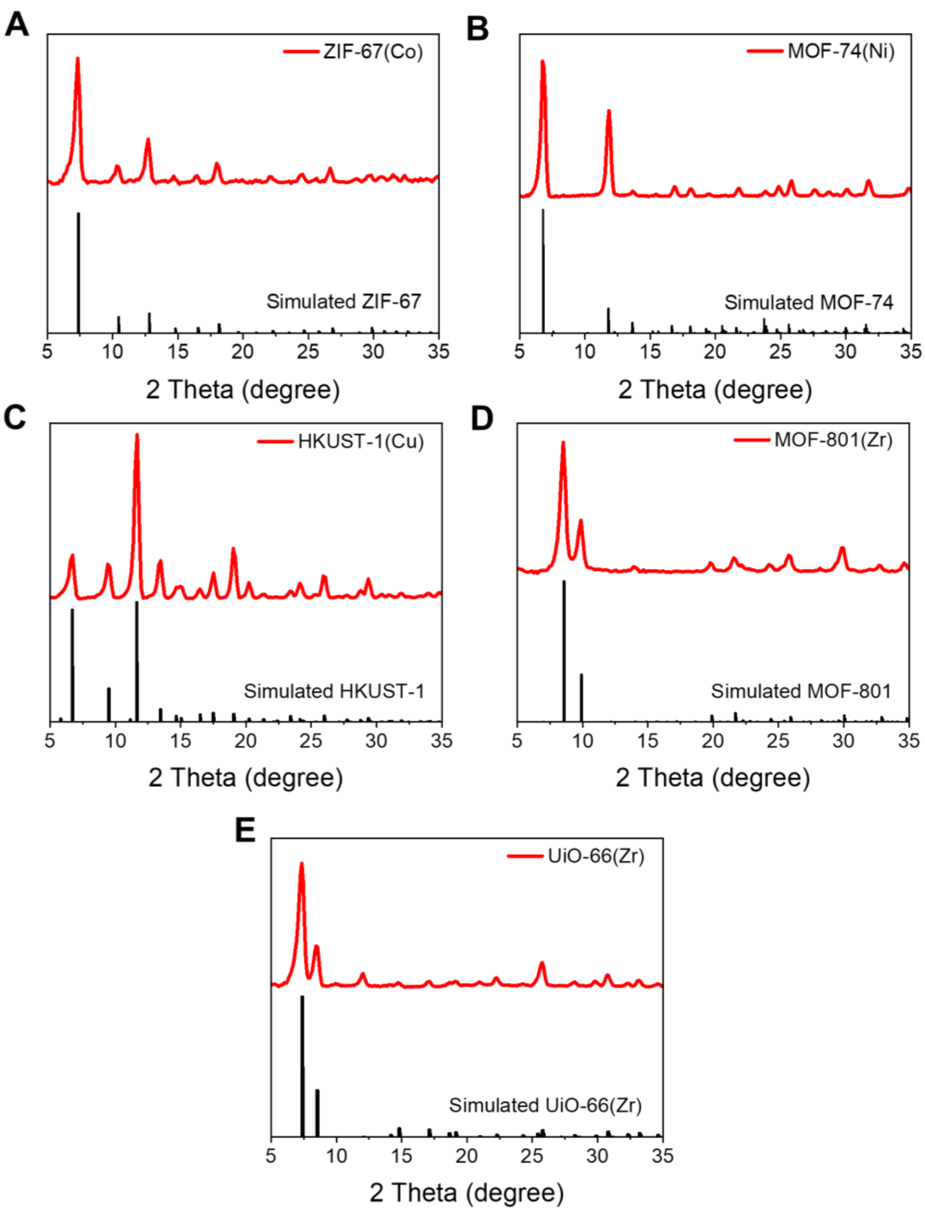

Figure S2. XRD patterns of the MOFs. (**A**) ZIF-67(Co), (**B**) MOF-74(Ni, (**C**) HKUST-1(Cu), (**D**) MOF-801(Zr), and (**E**) UiO-66(Zr).

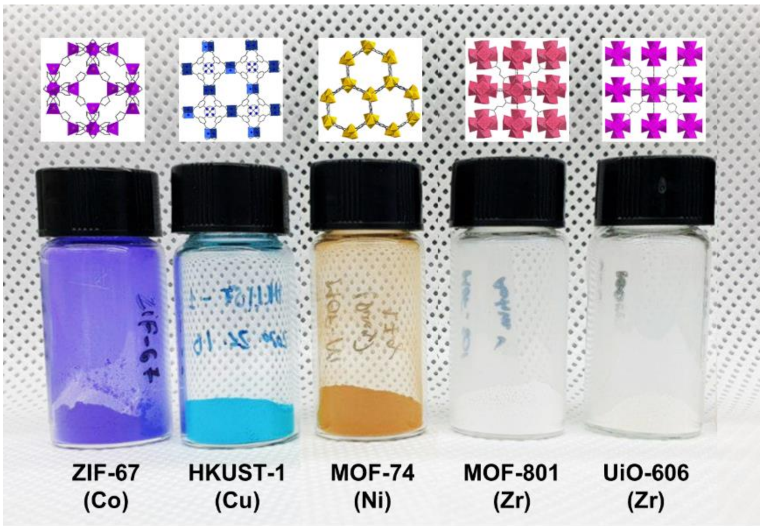

Figure S3. A digital photo of the five commonly used MOFs.

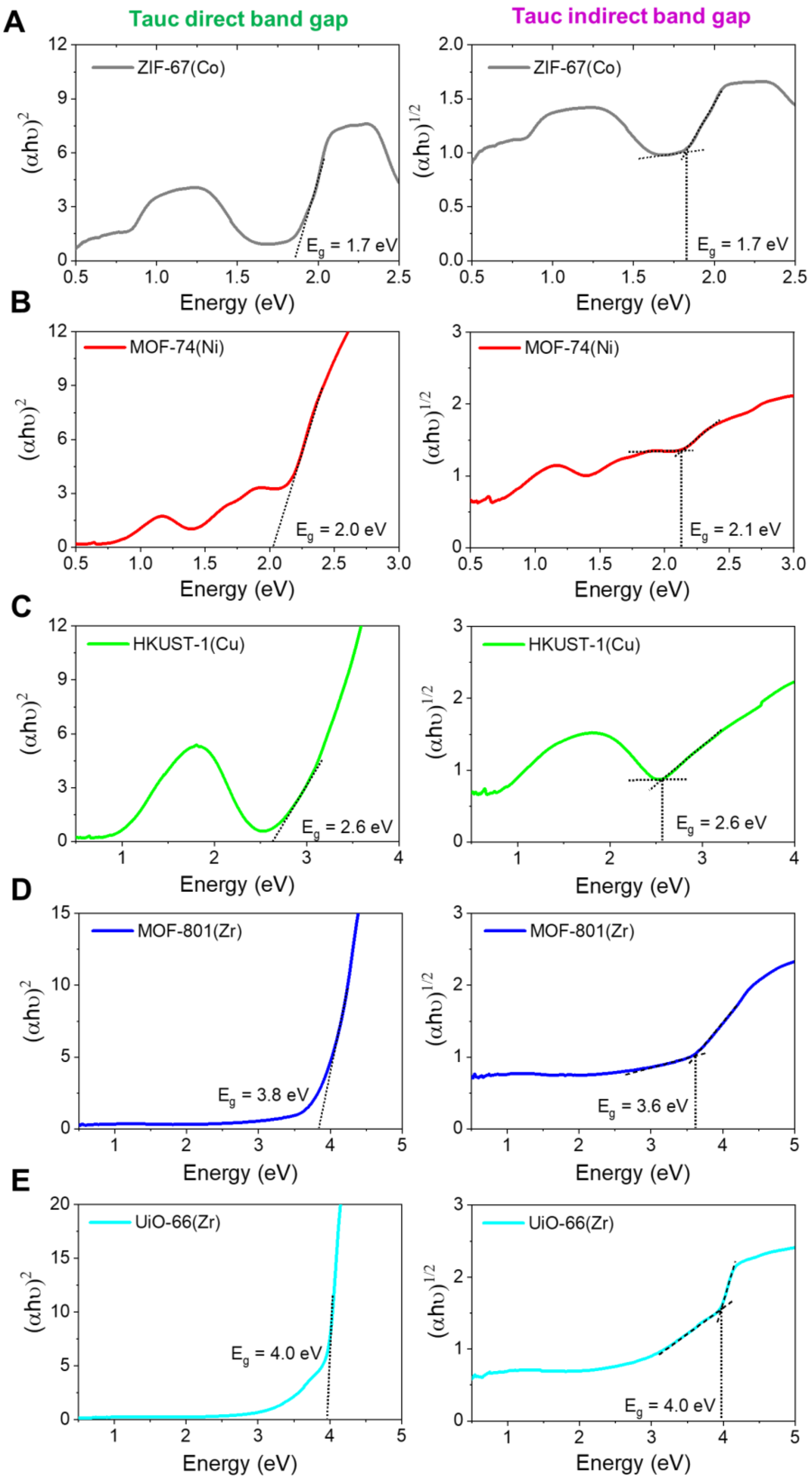

Figure S4. Tauc plots of the MOFs. α is the absorption coefficient, h is Planck's constant, and *v* is the photon's frequency. From left to right are Tauc direct band gap without baseline correction and indirect band gap with baseline correction for (A) ZIF-67(Co), (B) MOF-74(Ni), (C) HKUST-1(Cu), (D) MOF-801(Zr), and (E) UiO-66(Zr).

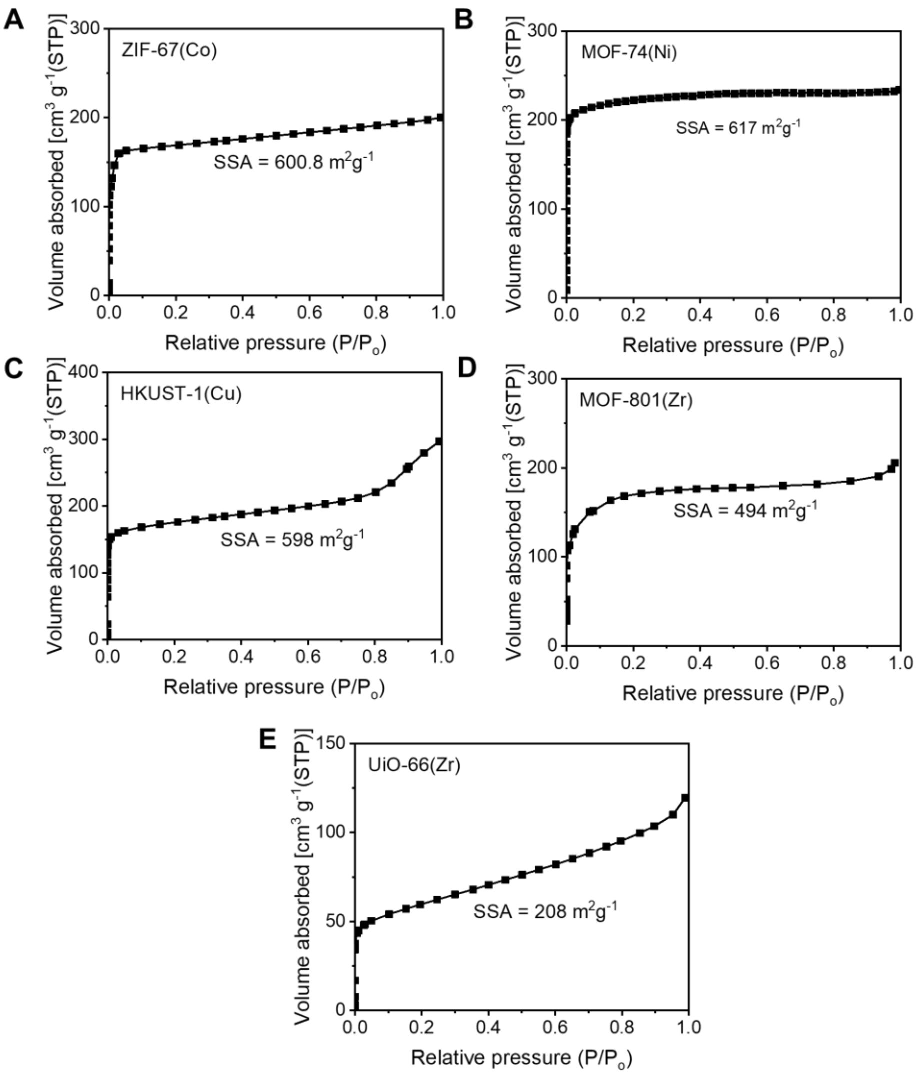

Figure S5. Adsorption isotherm showing nanoporous characteristics of the MOFs. (**A**) ZIF-67(Co), (**B**) MOF-74(Ni, (**C**) HKUST-1(Cu), (**D**) MOF-801(Zr), and (**E**) UiO-66(Zr).

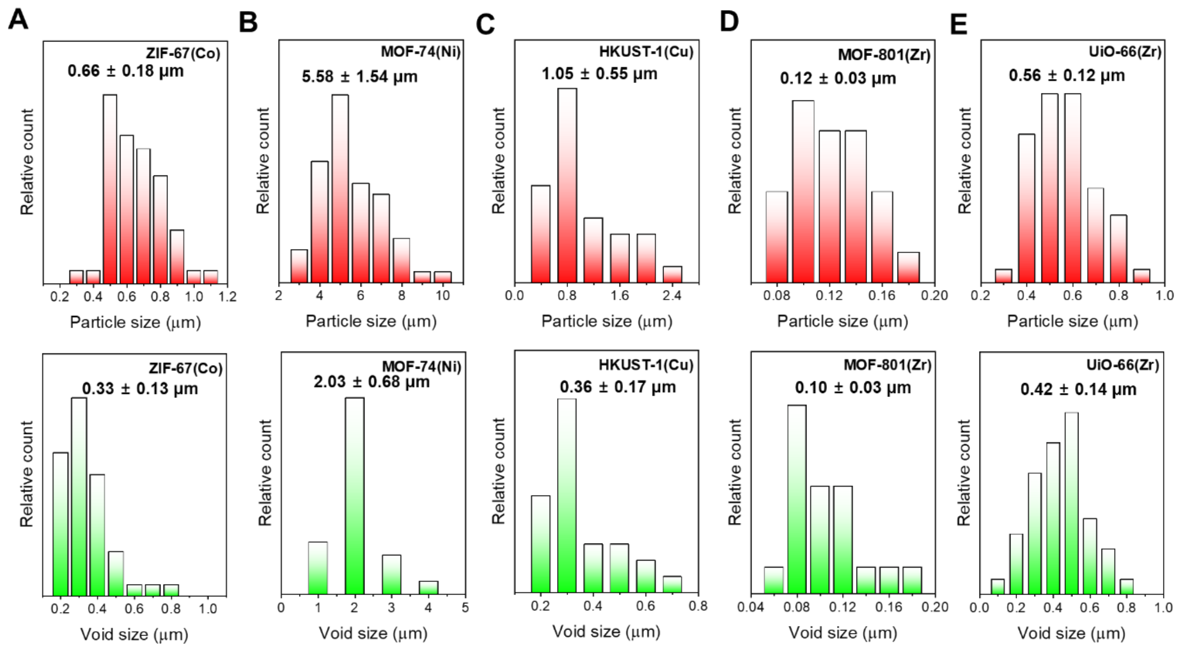

Figure S6. Morphological particle and void size distributions in the MOFs. From top to bottom are the particle size and void size distributions of (**A**) ZIF-67(Co), (**B**) MOF-74(Ni, (**C**) HKUST-1(Cu), (**D**) MOF-801(Zr), and (**E**) UiO-66(Zr). The particle and void size were measured to be 0.66 ± 0.18 µm and 0.33 ± 0.13 µm for ZIF-67(Co), 5.58 ± 1.54 µm and 2.03 ± 0.68 µm for MOF-74(Ni), 1.05 ± 0.55 µm and 0.36 ± 0.17 µm for HKUST-1(Cu), 0.12 ± 0.03 µm and 0.10 ± 0.03 µm for MOF-801(Zr), and 0.56 ± 0.12 µm and 0.42 ± 0.14 µm for UiO-66(Zr), respectively.

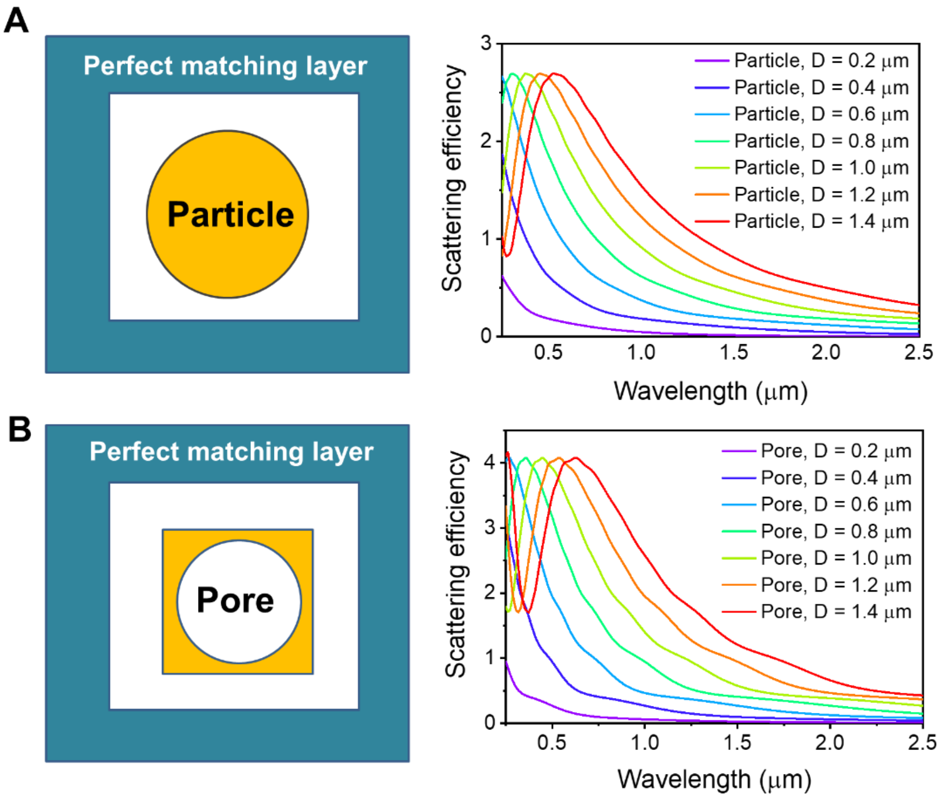

Figure S7. FDTD simulations for UiO-66(Zr) with different particle and void sizes. From left to right, (**A**) simulation model and scattering efficiency at different particle sizes, (**B**) simulation model and scattering efficiency at different void sizes.

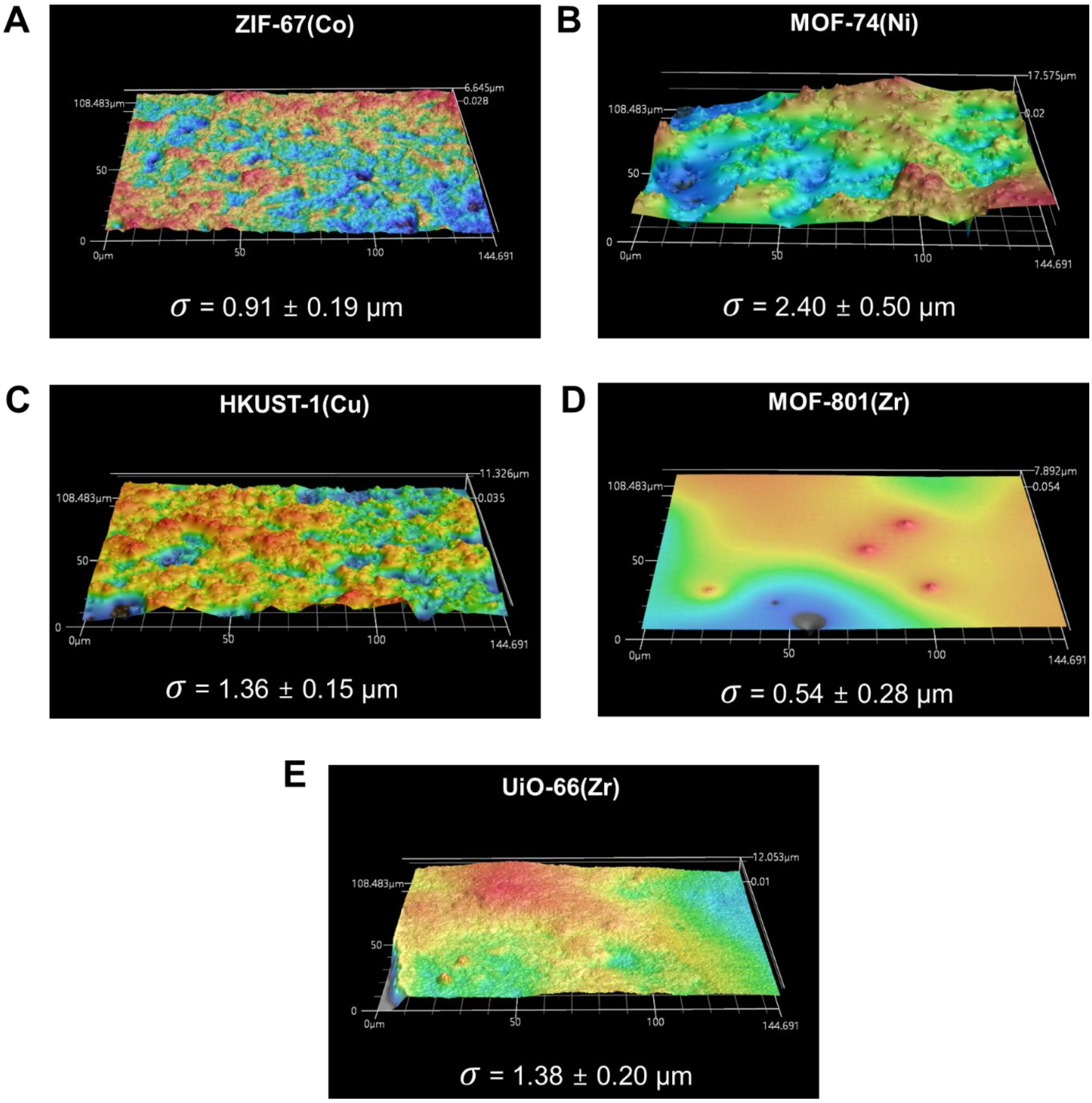

Figure S8. The surface roughness (σ) of the MOF films measured by a confocal microscope. (**A**) ZIF-67(Co), (**B**) MOF-74(Ni, (**C**) HKUST-1(Cu), (**D**) MOF-801(Zr), and (**E**) UiO-66(Zr).

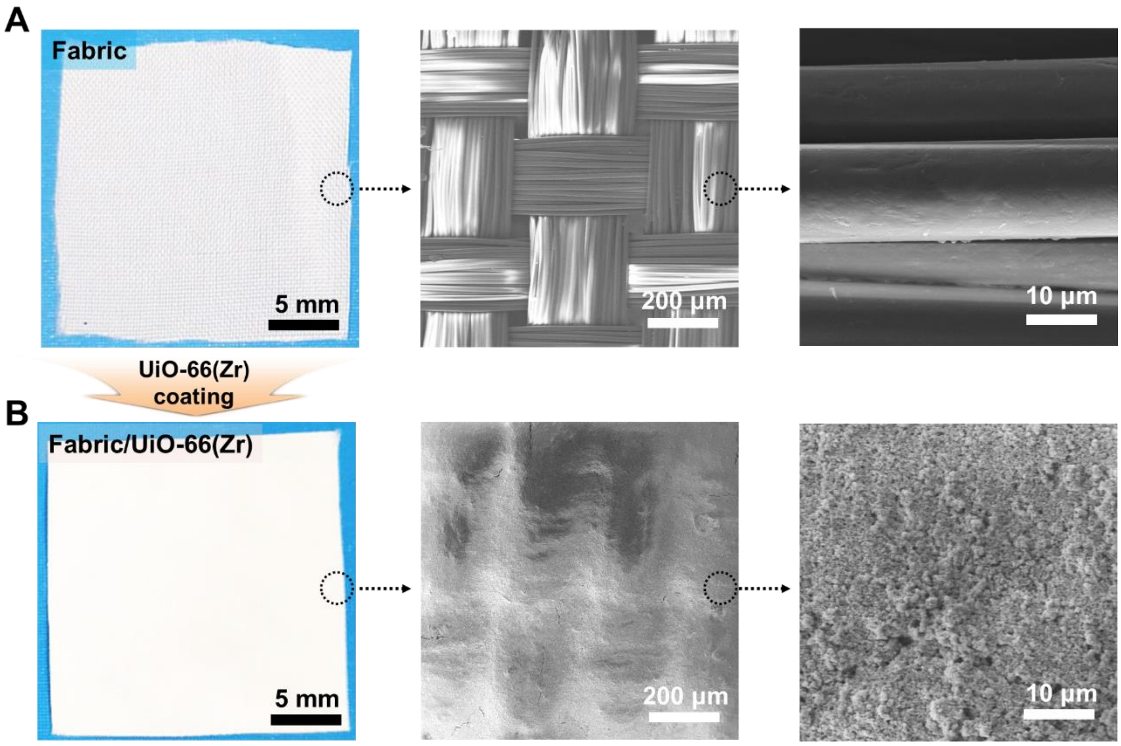

Figure S9. Morphological characteristics of the polyester fabric before and after UiO-66(Zr) painting. From left to right, digital image, low- and high-magnification SEM images of (**A**) fabric and (**B**) fabric/UiO-66(Zr), respectively.

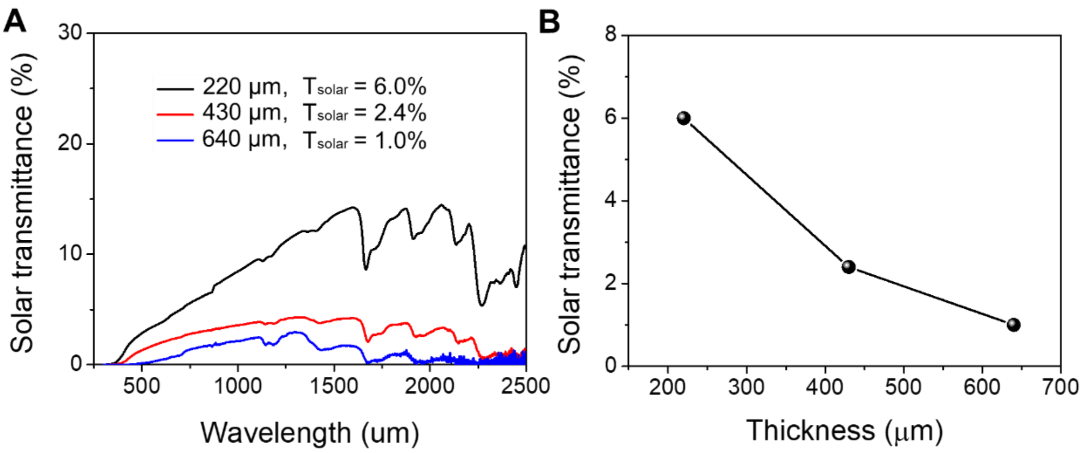

Figure S10. Correlation between solar transmittance and thickness of the fabric/UiO-66(Zr)-based emitter. (**A**) Wavelength-dependent solar transmittance of different thicknesses of the cooler. (**B**) Thickness-dependent solar transmittance of the cooler.

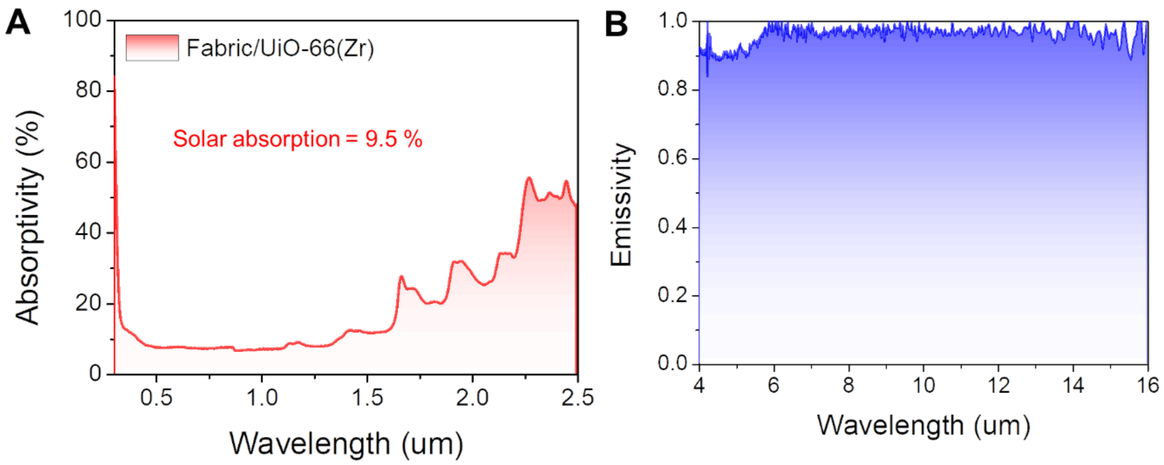

Figure S11. Optical properties of the fabric/UiO-66(Zr)-based emitter. (**A**) Solar absorptivity, (**B**) IR emissivity.

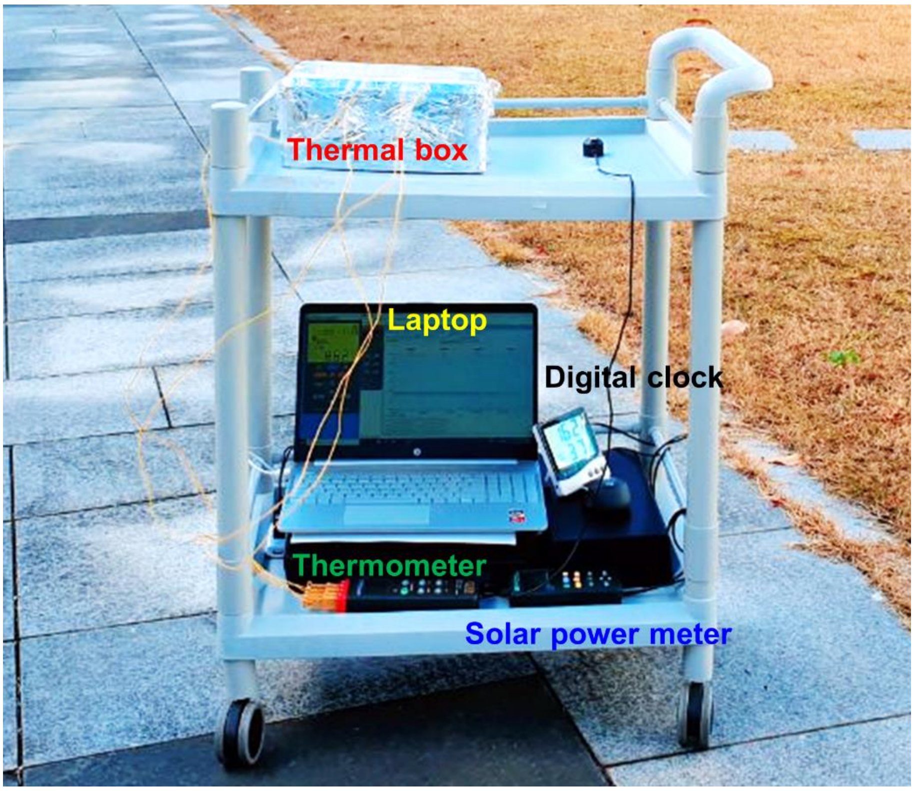

Figure S12. Digital photo showing the experimental apparatus for outdoor PDRC experiments.

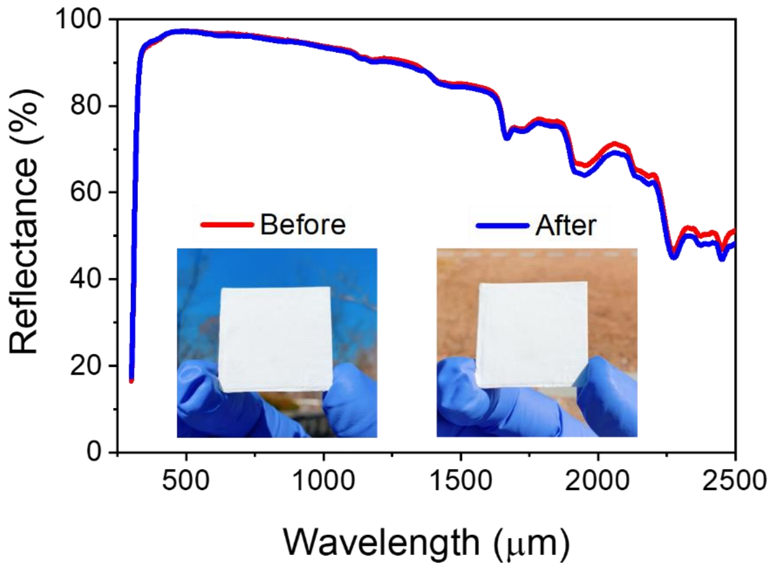

Figure S13. UV stability of the UiO-66(Zr)-based cooler before and after sunlight exposure for a week. Insets are the pictures of the cooler before and after the test.

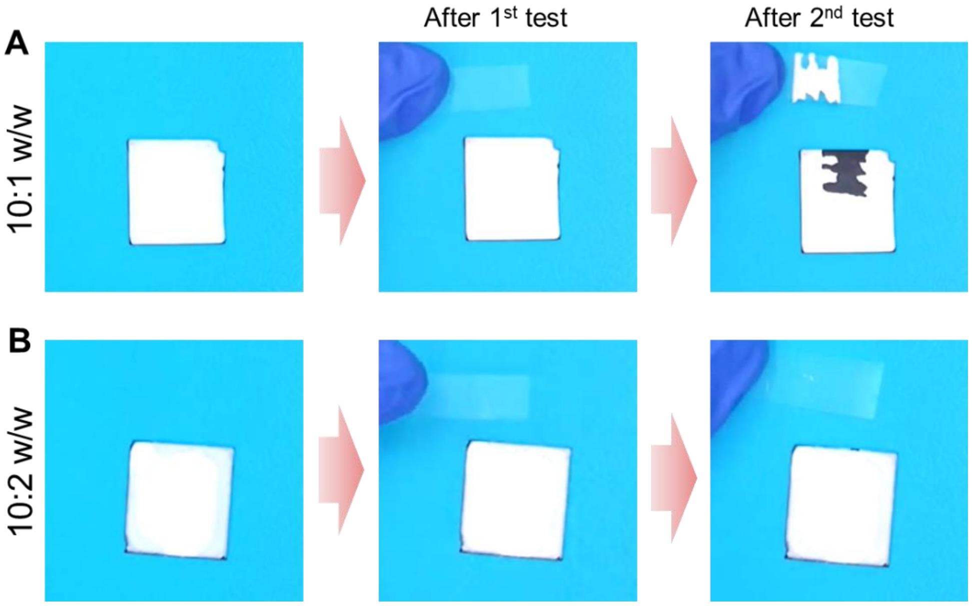

Figure S14. Tape pull-off tests for UiO-66(Zr) films on Si substrate with different MOF to PVDF binder ratios. (A) 10:1 w/w. **(B)** 10:2 w/w. The pull-off tests were conducted using a 3M scotch tape. The adhesion stability of the films was observed to depend on the binder content. The higher binder content generally resulted in improved adhesion to the Si substrate.

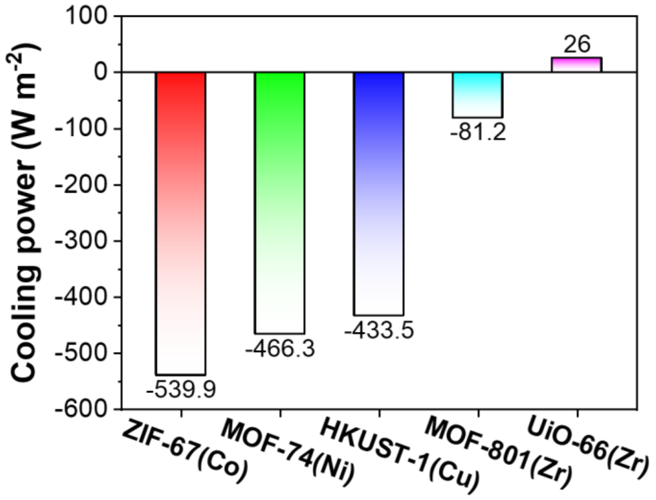

Figure S15. Theoretical cooling powers of the five MOFs.

Table S1. Summary of previously reported PDRC emitters

**Ref.** (#)	**Material design** [Top-to-bottom]	**Solar reflectance** [%]	**Mid-IR emissivity** [%]	**Cooling power** [W m^{-2}]	**Cooling temperature** [°C]
**This work**	**UiO-66(Zr)/fabric**	**91.5**	**96.8**	**26**	**5**
S6	Multilayer $Hf_2O/SiO_2/Ag$	97	50 – 80	40	5
S7	Pyramid PDMS	95	98	20	6.2
S8	$SiO_2/Si_3N_4/Al_2O_3/Ag$	95	87	66	8.2
S9	Polyethylene aerogel	92.2	79.9	96	13
S10	Hierarchically structured PMMA	95	98	85	8.9
S11	PAN nanofibers with beads	95	>70	96	3
S12	Hierarchically porous P(VdF-HFP)	96	97	96	6
S13	SiO2@polymethylpentene/Ag	96	93	93	8
S14	Expanded PTFE/Ag	98	90	120.6	2.7
S15	Nano-micro-structured plastics	96	90	100	7.5

Table S2. Synthesis conditions of different MOFs

MOF type	Metal salt	Metal salt solvent	Ligand	Ligand solvent	Synthesis conditions
**ZIF-67(Co)**	$Co(NO_3)_2\cdot6H_2O$	EtOH	2-Methyl imidazole<br>$CH_3C_3H_2N_2H$	EtOH	140 °C/24h
**MOF-74(Ni)**	$Ni(NO_3)_2\cdot6H_2O$	DEF	2,5-Dihydroxyterephalic acid<br>$C_6H_3(OH)_2(COOH)_2$	EG	130 °C/24h
**HKUST-1(Cu)**	$Cu(NO_3)_2\cdot3H_2O$	H_2O	Trimesic acid<br>$C_6H_3(COOH)_3$	EtOH	80 °C /20h
**MOF-801(Zr)**	$ZrCl_4$	DMF	Fumaric acid<br>$HO_2CHC{=}CHCO_2H$	DMF	120 °C/24h
**UiO-66(Zr)**	$ZrCl_4$	DEF	Terephthalic acid<br>$C_6H_4(COOH)_2$	DEF	135 °C/48h

Supporting references

(S1) Zhang, Q.; Wang, S.; Wang, X.; Jiang, Y.; Li, J.; Xu, W.; Zhu, B.; Zhu, J. Recent Progress in Daytime Radiative Cooling: Advanced Material Designs and Applications. *Small Methods* **2022**, *6*, 2101379. https://doi.org/10.1002/smtd.202101379.

(S2) Huang, Y.; Tao, C. A.; Chen, R.; Sheng, L.; Wang, J. Comparison of Fabrication Methods of Metal-Organic Framework Optical Thin Films. *Nanomaterials* **2018**, *8*, 676. https://doi.org/10.3390/nano8090676.

(S3) Connolly, B. M.; Aragones-Anglada, M.; Gandara-Loe, J.; Danaf, N. A.; Lamb, D. C.; Mehta, J. P.; Vulpe, D.; Wuttke, S.; Silvestre-Albero, J.; Moghadam, P. Z.; Wheatley, A. E. H.; Fairen-Jimenez, D. Tuning Porosity in Macroscopic Monolithic Metal-Organic Frameworks for Exceptional Natural Gas Storage. *Nat. Commun.* **2019**, *10*, 2345. https://doi.org/10.1038/s41467-019-10185-1.

(S4) Zhong, H.; Zhang, P.; Li, Y.; Yang, X.; Zhao, Y.; Wang, Z. Highly Solar-Reflective Structures for Daytime Radiative Cooling under High Humidity. *ACS Appl. Mater. Interfaces* **2020**, *12*, 51409–51417. https://doi.org/10.1021/acsami.0c14075.

(S5) Mandal, J.; Fu, Y.; Overvig, A. C.; Jia, M.; Sun, K.; Shi, N. N.; Zhou, H.; Xiao, X.; Yu, N.; Yang, Y. Hierarchically Porous Polymer Coatings for Highly Efficient Passive Daytime Radiative Cooling. *Science* **2018**, *362*, 315–319. https://doi.org/10.1126/science.aat9513.

(S6) Raman, A. P.; Anoma, M. A.; Zhu, L.; Rephaeli, E.; Fan, S. Passive Radiative Cooling below Ambient Air Temperature under Direct Sunlight. *Nature* **2014**, *515*, 540–544. https://doi.org/10.1038/nature13883.

(S7) Lee, E.; Luo, T. Black Body-like Radiative Cooling for Flexible Thin-Film Solar Cells. *Sol. Energy Mater. Sol. Cells* **2019**, *194*, 222–228. https://doi.org/10.1016/j.solmat.2019.02.015.

(S8) Chae, D.; Kim, M.; Jung, P. H.; Son, S.; Seo, J.; Liu, Y.; Lee, B. J.; Lee, H. Spectrally Selective Inorganic-Based Multilayer Emitter for Daytime Radiative Cooling. *ACS Appl. Mater. Interfaces* **2020**, *12*, 8073–8081. https://doi.org/10.1021/acsami.9b16742.

(S9) Leroy, A.; Bhatia, B.; Kelsall, C. C.; Castillejo-Cuberos, A.; Di Capua, M. H.; Zhao, L.; Zhang, L.; Guzman, A. M.; Wang, E. N. High-Performance Subambient Radiative Cooling Enabled by Optically Selective and Thermally Insulating Polyethylene Aerogel. *Sci. Adv.* **2019**, *5*, eaat9480. https://doi.org/10.1126/sciadv.aat9480.

(S10) Wang, T.; Wu, Y.; Shi, L.; Hu, X.; Chen, M.; Wu, L. A Structural Polymer for Highly Efficient All-Day Passive Radiative Cooling. *Nat. Commun.* **2021**, *12*, 365. https://doi.org/10.1038/s41467-020-20646-7.

(S11) Kim, H.; McSherry, S.; Brown, B.; Lenert, A. Selectively Enhancing Solar Scattering for Direct Radiative Cooling through Control of Polymer Nanofiber Morphology. *ACS Appl. Mater. Interfaces* **2020**, *12*, 43553–43559. https://doi.org/10.1021/acsami.0c09374.

(S12) Mandal, J.; Fu, Y.; Overvig, A. C.; Jia, M.; Sun, K.; Shi, N. N.; Zhou, H.; Xiao, X.; Yu, N.; Yang, Y. Hierarchically Porous Polymer Coatings for Highly Efficient Passive Daytime Radiative Cooling. *Science* **2018**, *362*, 315–319. https://doi.org/10.1126/science.aat9513.

(S13) Zhai, Y.; Ma, Y.; David, S. N.; Zhao, D.; Lou, R.; Tan, G.; Yang, R.; Yin, X. Scalable-Manufactured Randomized Glass-Polymer Hybrid Metamaterial for Daytime Radiative Cooling. *Science* **2017**, *355*, 1062–1066. https://doi.org/10.1126/science.aai7899.

(S14) Chae, D.; Lim, H.; So, S.; Son, S.; Ju, S.; Kim, W.; Rho, J.; Lee, H. Spectrally Selective Nanoparticle Mixture Coating for Passive Daytime Radiative Cooling. *ACS Appl. Mater. Interfaces* **2021**, *13*, 21119–21126. https://doi.org/10.1021/acsami.0c20311.

(S15) Gao, W.; Lei, Z.; Wu, K.; Chen, Y. Reconfigurable and Renewable Nano-Micro-Structured Plastics for Radiative Cooling. *Adv. Funct. Mater.* **2021**, *31*, 2100535. https://doi.org/10.1002/adfm.202100535.
